# Splenomegaly and Response to Splenectomy in Immune Thrombocytopenia

**DOI:** 10.3390/jcm13133712

**Published:** 2024-06-26

**Authors:** Emma Rabinovich, Kith Pradhan, Iffath Islam, Helen Tracy Davido, Radhika Gali, Peter Muscarella, Henny H. Billett

**Affiliations:** 1 Department of Medicine, Montefiore Medical Center, Albert Einstein College of Medicine, New York, NY 10461, USA; kith.pradhan@einsteinmed.edu (K.P.); hbillett@montefiore.org (H.H.B.); 2 Department of Surgery, Montefiore Medical Center, Albert Einstein College of Medicine, New York, NY 10461, USA; hdavido@montefiore.org; 3 Department of Surgery, Niagara Falls Memorial Medical Center, Niagara Falls, NY 14301, USA; peter.muscarella@nfmmc.org

**Keywords:** immune thrombocytopenia, ITP, splenectomy, splenomegaly

## Abstract

**Background**: Immune thrombocytopenia (ITP) is an acquired autoimmune disorder affecting patients of all ages and backgrounds. While current standards favor medical therapy in the frontline setting, splenectomy remains an integral part of treatment in refractory cases. Ideal parameters for patient selection for surgery remain elusive. **Methods**: Data for 40 adult patients undergoing splenectomy for ITP at a large urban center between 1 January 2010 and 1 July 2021 were collected and analyzed. **Results**: Most patients underwent uneventful laparoscopic splenectomy (95%). Complete or partial response at the time of last follow-up occurred in most patients (92.5%), with 60.0% requiring no additional medical therapy following surgery. Thrombosis was the predominant adverse event and the leading cause of death for two patients. Age and presence of splenomegaly appear to be associated with response to splenectomy. **Conclusions**: Splenectomy remains an effective therapy for selected patients with ITP. Predictors of positive response to splenectomy, such as younger age and the presence of splenomegaly, may help inform clinicians during patient selection for therapy. With strict attention paid to postoperative thromboprophylaxis, the diminishing use of splenectomy may not be warranted.

## 1. Introduction

Immune thrombocytopenia (ITP) is an acquired autoimmune disorder resulting in isolated thrombocytopenia and, in adults, characterized by relapses and recurrences [1]. First-line therapy for ITP consists of corticosteroids and intravenous immunoglobulin [2,3]. Second-line treatments, including rituximab and thrombopoietin receptor agonists (TPO-RA), provide similarly high initial response rates of 80–90% [4,5,6,7], but maintaining platelet counts without continued therapy remains highly challenging. Sustained response rates following discontinuation of therapy are only approximately 20% with rituximab and 10–30% with TPO-RA [8,9]. Patients maintained on TPO-RA have had sustained response rates of up to 70% at 6 months, but vein thrombosis rates are increased with these agents, and annualized thrombosis rates appear to be 2–3 times higher in patients treated with TPO-RA than in other ITP patients [5,7]. Even fostamatinib, recently approved for refractory ITP, has an overall response of just 43%, though without a notable increase in thrombosis risk [10,11].

Splenectomy was considered an integral part of second-line ITP treatment until the inception of the newer agents described above [2,12,13]. Since the widespread use of these agents, the role and timing of splenectomy has become an area of debate [14]. Whether splenectomy should remain an important treatment option for younger patients or for those refractory to multiple lines of medical therapy is unclear [14]. By reviewing a wide range of patient characteristics and newer medical and surgical outcomes, this study aims to examine preoperative and postoperative practices in patient evaluation and management to assess and optimize treatment outcomes in a diverse population.

## 2. Methods

### 2.1. Patient Selection

This is a retrospective cohort study of patients of adult patients who underwent splenectomy for treatment of ITP at Montefiore Medical Center between January 2010 and July 2021. Pediatric patients were excluded, as the natural history of childhood ITP differs from adult forms, often following a more benign and self-limited course [15]. Patients were identified with an interactive software application that integrates clinical and administrative datasets using search diagnosis codes for splenectomy and ITP. Individual patient chart reviews excluded those whose primary indication for splenectomy was not ITP. Patients without prior available medical records or follow-up data, typically tertiary referrals, were also excluded.

### 2.2. Data Collection

Data collected during chart review consisted of basic patient characteristics, including age, race, and gender, as well as variables of interest relating to pre-existing conditions, prior lines of therapy, and management decisions regarding anticoagulation and vaccination. Response to prior lines of medical treatment with steroids, intravenous immunoglobulin (IVIG), TPO-RA, and immunosuppressive agents was verified by a concordant rise in platelet count. The time frame for determining treatment response was flexible by design, given the known variability in response times to different therapies. The response was based on first exposure to each treatment type. Imaging methods prior to splenectomy were also recorded. The presence of splenomegaly was recorded according to radiographic interpretation, as height and sex can alter considerations regarding the upper limit of normal. Both minimally and grossly enlarged spleens were included under this designation. Bone marrow biopsy results were reviewed. Surgical complications were assessed according to the Clavien–Dindo classification [16]. Rates of postoperative superior mesenteric vein thrombosis or portal vein thrombosis, pancreatic fistula formation, and length of stay were documented. Due to patient data collection limitations prior to the adaptation of electronic medical records (EMRs), relapses were not collected for patients who underwent splenectomy before the EMR transition in 2015.

Primary outcome data consisted of treatment response one week after surgery, thirty days after surgery, and at last follow-up in order to understand both rapid benefit as well as long-term efficacy. Responses were assessed by both absolute platelet count and response category. Complete response (CR) was defined as platelet count ≥ 100 × 10^9^/L and the absence of bleeding [1,3]. Partial response (PR) was defined as a platelet count between 30 and 100 × 10^9^/L and the absence of bleeding [1,3]. No response (NR) was defined as failure to attain a platelet count of ≥30 × 10^9^/L or the presence of bleeding [1,3]. Using a two-fold increase from baseline was avoided due to inconsistencies in baseline counts across patients. Patients who were off all medical therapy at last follow-up were subcategorized into those who never received any further medical therapy following surgery and those who transiently required additional therapy but were ultimately able to discontinue all treatment for at least two years. Overall mortality and last follow-up were assessed on 5 January 2022.

### 2.3. Statistical Analysis

Associations between variables were examined with a Kendall–Tau correlation coefficient, a Fisher test of proportions, or a Kruskal–Wallis test, depending on whether the variables were both numeric, both categorical, or numeric/categorical, respectively. A multivariable analysis of the ranked responses at last follow-up was performed using an ordinal logistic regression model to examine relationships with various clinical variables while accounting for age at splenectomy. A Brant’s test validated the assumption of proportional odds on each of the variables included in the model. All analysis was done in R (version 4.2.1) using the MASS package [17].

### 2.4. Institutional Review

Approval for this study was obtained from the institutional review board at Albert Einstein College of Medicine. Patient consent was waived due to the retrospective nature of this study.

## 3. Results

Ninety-five patients underwent splenectomy at Montefiore between 1 January 2010 and 1 July 2021. Of these, manual chart review eliminated fifty-five patients who had multiple indications for splenectomy, did not have any pre- or postoperative data, or were under age 18 at the time of splenectomy. All remaining 40 patients were included. Of these 40 patients, 29 patients were accrued after Montefiore had transitioned to electronic medical records (EMRs) in 2015 [18].

Demographic and Preoperative Clinical Data. Demographic and clinical data on accrued patients is summarized in Table 1. The patient population was 60% female with an average age of 46.6 years (range 19.6–92.6) at the time of splenectomy. Our multiethnic cohort included 16 (40.0%) Hispanic, 10 (25.0%) Black, 7 (17.5%) White, and 1 (2.5%) Asian patient, which is representative of the population residing in our treatment area. Co-morbid autoimmune disorders were found in 35% of patients. Preoperative imaging was completed in most patients (*n* = 32, 80%), with computed tomography (*n* = 19) being the most common modality, followed by ultrasound (*n* = 12) and MRI (*n* = 1). Splenomegaly was described in approximately one-third of the patients with imaging (*n* = 12, 37.5%). Just over half (*n* = 21, 52.5%) underwent bone marrow biopsy prior to splenectomy to exclude an alternative diagnosis.

Medical treatment practices and outcomes. Initial treatment consisted of steroids and IVIG (or Rho(D) immunoglobulin), in concert and/or sequentially, for most patients (*n* = 38, 95%). Due to differences in practice between providers, subsequent presentations were also treated with one or both treatments in the acute setting based on physician preference. Of 30 patients who received IVIG and steroids in concert, 24 patients (80%) had a response. Of 32 patients who received a trial of steroid monotherapy, 19 (59.4%) responded. Of the 18 patients who received a trial of IVIG monotherapy, 13 (72.2%) had a response. Five patients failed to respond to any combination of IVIG and/or steroids.

Second-line treatment was highly variable among providers. Deciding factors included treatment setting, insurance coverage, and patient preference. Following relapse on initial treatment, therapy consisted of TPO-RA (*n* = 11, 27.5%); rituximab (*n* = 12, 30%); danazol (*n* = 5, 12.5%); and immunosuppressive therapy (*n* = 1, 2.5%). The median number of unique lines of therapy was 2 (range 0–5) across all patients. The median time from diagnosis to splenectomy was 1 year (range 0–11) in these patients. Relapses were evaluated in the 29 patients who were treated following the transition to EMRs, as earlier data were incomplete. The median number of relapses in these patients was 3 (range 0–16).

Surgical approach and complications. The vast majority of patients underwent laparoscopic splenectomy. Only two patients (5%) required elective open splenectomy due to a history of prior emergency laparotomy and a need for concurrent surgical operation. No unexpected findings of lymphoproliferative disorder or other hematologic malignancy were found on pathology from any resected specimens. Although most patients (*n* = 30, 75%) were discharged without complications (Clavien–Dindo grade 0), postoperative complications occurred in 10 patients (25%). Of these ten patients, the majority had Clavien–Dindo grade 1 (*n* = 3, 7.5%) or grade 2 (*n* = 6, 15%) findings. The median length of stay (LOS) was 5 days (range 1–121 days). Three patients developed superior mesenteric or portal vein thrombosis within 90 days of surgery. No instances of overwhelming post-splenectomy infection or pancreatic fistula formation were noted. These data are detailed in Table 1.

Splenectomy outcomes. Platelet counts in the initial week following surgery (±3 days) were highly variable, with persistent thrombocytopenia in some patients and reactive thrombocytosis in others (median platelet count 328 × 109/L, range 15–1265). Of the four patients whose first-week platelet counts remained low, three eventually required additional medical therapy, and one died within 30 days. Of the 10 patients with thrombocytosis, 8 never required additional medical therapy, while 2 required transient medical therapy but ultimately achieved complete response without medical therapy.

Table 2 summarizes patient outcomes. The median platelet count was 221 × 10^9^/L (range 3–609) at 30 days. Most patients had achieved a complete (*n* = 30, 75%) or partial (*n* = 2, 5%) response. Six patients (15%) had no response to surgery. Two patients died (*n* = 5%) within thirty days.

The median platelet count at last follow-up, at a median time of 3.91 years, was 263 × 109/L (range 8–766). Thirty-seven (93%) patients had a complete (*n* = 34, 85%) or partial (*n* = 3, 8%) response either on or off medical therapy at the time of last follow-up. Of those with a complete or partial response, the majority (*n* = 24, 60%) remained entirely off medical therapy following surgery. Five patients (12.5%) transiently required medical therapy after splenectomy but remained in remission without additional medical therapy for more than two years. Nine (22.5%) patients remained on medical therapy at the time of last follow-up. Of nine patients on medical therapy, only one patient remained severely thrombocytopenic post-splenectomy with no response at the time of last follow-up, though this was a very short follow-up, with only three months post-surgery data.

Overall mortality. Two patients died within 30 days of surgery, on postoperative days (POD) 21 and 30, both from pulmonary emboli. One patient had had highly refractory ITP complicated by intracranial bleeding, precluding any prophylactic anticoagulation during his hospitalization. After undergoing splenic embolization prior to splenectomy without meaningful improvement, he then underwent expedited splenectomy. Following a failure to improve after splenectomy, the patient resumed TPO therapy with a significant rise in his platelet count and then suffered a lethal pulmonary embolism.

The second patient was initially admitted for recurrent acute pancreatitis and requested elective splenectomy during his hospitalization to facilitate discontinuation of chronic steroid use. A bone marrow biopsy during this admission was consistent with a diagnosis of ITP. The splenectomy was performed one week into his hospitalization, and he was discharged 9 days later to a rehabilitation facility with a normal platelet count. He died at home two weeks later. On autopsy, pulmonary emboli were determined to be the cause of death. Prothrombin G20210A variant heterozygosity was also a contributing cause of death. Anticoagulation had been deferred due to severe anemia in the setting of acute illness, which was pending further evaluation. The patient did not have a history of prior thrombotic events, and a pathology review of the spleen was unremarkable.

Predictors of Splenectomy Outcome. A summary of the association of demographic, preoperative, and postoperative data with outcomes at last follow-up is presented in Table 3. No significant differences were seen at any point in treatment across patients of different races, blood types, or history of antiphospholipid syndrome (APLS), HIV, or other comorbid conditions. Response to IVIG or to IVIG and steroids was predictive of 7-day post-splenectomy response (*p* = 0.011 and 0.0035, respectively) but not of 30-day or last follow-up response after surgery. Bone marrow confirmation prior to splenectomy did not significantly correlate with improved splenectomy outcomes. Neither race nor ethnicity correlated with differences in outcome.

Spleen Size. Of 32 patients who had available preoperative imaging, 12 patients (40%) had evidence of splenomegaly. Following splenectomy, 10 of these 12 patients (83%) had complete responses without any further treatment, and the remaining 2 patients were able to successfully stop medical therapy for greater than two years at last follow-up (Table 3). Those patients who underwent splenectomy without evidence of splenomegaly on imaging displayed a variety of responses, with eight patients (44%) remaining on medical therapy at the time of last follow-up. The difference in outcomes between the two groups was found to be significant (*p* = 0.018), even as early as 7 days (*p* = 0.040).

Total Lines of Therapy and Relapses. Wide variation in the number of treatments used before splenectomy was noted. For 24 patients who achieved a complete response and required no further medical therapy at last follow-up, the median number of treatments was two. For nine patients who remained on medical therapy at the time of last follow-up, the median was four. Patients who required fewer lines of treatment appeared to do significantly better after splenectomy (*p* = 0.0065). An inverse correlation was seen between high rates of relapse and platelet counts at 30 days (*p* = 0.017).

Age at Splenectomy. The median age for the entire cohort was 41.2 years (range 19.6 to 92.6 years). There was significant variation in median age according to outcome at last follow-up (*p* = 0.034): the median age for patients who achieved complete response and required no further medical therapy at last follow-up was 37.1 years, whereas the median ages for those requiring further medical therapy either transiently or persistently at last follow-up were 62.5 and 55.8 years, respectively.

## 4. Discussion

Meta-analyses have demonstrated a 60–70% complete response rate to splenectomy [19]. Though no clear preoperative characteristics that predict splenectomy response have previously been identified, there are some data demonstrating better outcomes in patients <50 years of age and those responsive to first-line medical therapy [20]. Others have shown no statistically significant association between age, gender, body mass index, ASA classification, disease duration, accessory spleens, splenic weight, conversion to open surgery, or perioperative complications with post-splenectomy response rates [21].

While offering patients a high rate of cure, splenectomy is also associated with increased rates of vein thrombosis and sepsis [22]. The risk of overwhelming infection appears to be greatest in the few months immediately after surgery but has been dramatically reduced by routine vaccination [22,23]. Length of stay and postoperative complications have been minimized with newer laparoscopic techniques, splenic embolization, and thromboprophylaxis [24,25,26]. The treatment of relapsed disease following splenectomy does not impact subsequent therapeutic choice. Post-splenectomy response has been demonstrated to be similar to that of non-splenectomized patients with refractory ITP. TPO-RA and rituximab have shown similarly high response rates, 70–80%, when given following splenectomy in those who relapsed after surgery [4,27]. Responses to fostamatinib were also seen in splenectomized patients [10].

Preoperative treatment in our cohort was highly individualized, but some clear practice patterns emerged. First-line treatment with a combination of steroids and/or IVIG was seen as the mainstay of management in accordance with society guidelines [14,28]. As evidence to inform splenectomy candidacy and timing remains limited, deferring surgical intervention at least 12 months from diagnosis appears to now be a generally accepted practice [29]. A trial of at least one second-line medical therapeutic option is also recommended [28,29]. Our study noted a significant association between the number of lines of treatment and relapses with outcomes at last follow-up, though it is unclear whether additional lines of therapy may affect response to splenectomy or if more refractory patients are also less likely to have durable responses with splenectomy.

The thirty-day mortality in our cohort exceeded previously reported rates for splenectomy in this setting. Retrospective data for splenectomy for various indications demonstrated overall mortality rates from 0–3.5% [30,31,32]. The two patients who died within thirty days of surgery both died of pulmonary emboli. A careful review of both patients’ histories revealed that both patients had a contraindication to prophylactic anticoagulation, which was thus deferred. One death was complicated by the addition of TPO-RA therapy and a contraindication to anticoagulation due to a recent cerebral bleed, while the other had active pancreatitis, a genetic thrombophilia, and a severe anemia of unclear etiology with a contraindication to anticoagulation.

Thrombosis is a well-established risk with splenectomy [33,34]. Though no prospective trials definitively outline the optimal thromboprophylaxis after splenectomy, several retrospective reviews have proposed that both early pharmacologic and extended thromboprophylaxis may significantly decrease rates of thrombosis [35,36]. The remaining operative outcomes and responses at our institution were overall in line with previous reports [14,15,25]. The majority of patients underwent uncomplicated laparoscopic splenectomy with short hospital stays. The thrombosis rate of 5% is similar to that of recent publications [14,25].

Identifying consistent markers to predict splenectomy outcomes has proven challenging due to variability in preoperative practices [16,17,18,25,30,37]. Given inconsistency across studies, preoperative evaluation remains at the discretion of the referring provider, with substantial variations in practice [38]. Only 80% of patients at our institution underwent abdominal imaging prior to surgery. Bone marrow biopsy to confirm diagnosis of ITP by excluding other causes of thrombocytopenia is widely utilized but also frequently avoided in patients deemed low risk for hematologic malignancy. Despite these limitations, several factors correlating with improved splenectomy outcomes were identified.

We show that patients with enlarged spleen size on imaging had significantly better outcomes following surgery. This trend was noted both early and late in the postoperative course. Other significant findings included better outcomes in patients who had received fewer lines of therapy. Increased age at the time of splenectomy also correlated with a worse response.

The prognostic significance of age has been previously reported and is validated in our cohort [20,39]. Other variables, consisting of spleen size and prior lines of therapy, have not been well characterized previously. Responsiveness to steroids and IVIG as a prognostic tool has been reported previously but did not reach long-term significance in our cohort [15,40].

Only one patient remained non-responsive to treatment following splenectomy, while nine in total remained on treatment. Though prior efficacy data for novel agents has raised concerns regarding patient response rates following splenectomy, second-line therapy following splenectomy was found to be generally effective in our cohort, either with continued or fixed-duration use [7]. The high rate of medical therapy discontinuation can be an additional consideration in patients concerned with financial toxicity that can be associated with novel agents.

## 5. Conclusions

Expert recommendations have generally moved in favor of additional medical treatment in second-line settings prior to proceeding with irreversible surgical intervention. Though highly efficacious, novel agents rituximab, TPO-RA, and fostamatinib all also have associated side effects, limitations, and potentially significant financial costs. Consequently, the diminishing use of splenectomy may not be warranted, as the response rate remains superior to these newer agents.

Our findings highlight important preoperative considerations, such as spleen size, as well as postoperative risks, including thrombosis, that may impact surgical outcomes. Improved understanding regarding optimal splenectomy candidates and risk mitigation measures to improve postoperative morbidity and mortality will be integral to maintaining the relevancy of splenectomy in the new ITP treatment landscape.

## Figures and Tables

**Table 1:** Cohort demographics, clinical findings, and pre- and postoperative data.

	Patients (*n* = 40)
Median Age, years (Range)	46.6 (19.6–92.6)
Female *n* (%)	25 (62.5)
**Race, *n* (%)**	
Hispanic	16 (40)
Black	10 (25)
White	7 (17.5)
Asian	1 (2.5)
Declined/Not specified	6 (15)
***Clinical Characteristics, n (%)***	
Comorbid Autoimmune Condition	14 (35)
Antiphospholipid Syndrome	2 (5)
HCV/HIV	3 (7.5)
**Preoperative Responsive to Therapies, *n* (%)**	
Steroids (*n* = 32)	19 (59.4)
IVIG (*n* = 18)	13 (72.2)
Steroids and IVIG (*n* = 30)	24 (80)
TPO (*n* = 11)	8 (72.7)
Rituximab (*n* = 12)	5 (41.7)
Danazol (*n* = 5)	3 (60)
Immunosuppressive (*n* = 1)	1 (100)
***Preoperative Evaluation, n (%)***	
Abdominal imaging	32 (80)
CT	19 (47.5)
US	12 (30)
MRI	1 (2.5)
Bone marrow biopsy	21 (52.5)
Preoperative vaccination	18 (45)
Splenomegaly (*n* = 32)	12 (37.5)
***Surgical Course, n (%)***	
Clavien–Dindo Grade Complications (>0)	10 (25)
Laparoscopic (vs. Open)	38 (95)
30-day mortality	2 (5)
PE	2 (5)
SMVT/PVT	3 (7.5)

**Table 2:** Response to splenectomy at POD 30 and last follow-up by platelet count.

	Patients (*n* = 40)
***Platelet Count at 30 Days, n (%)***	
Complete response (CR)	30 (75)
Partial response (PR)	2 (5)
No response (NR)	6 (15)
Deceased at POD 30	2 (5)
***Response at Last Follow-Up by Platelet Count, n (%)***	
Complete response (CR)	34 (85)
Partial response (PR)	3 (7.5)
No response (NR)	1 (2.5)
Deceased at POD 30	2 (5)
***Response at Last Follow-Up by Treatment, n (%)***	
No further medical therapy	24 (60)
Off medical therapy for >1 year	5 (12.5)
On medical therapy	9 (22.5)
Deceased at POD 30	2 (5)

**Table 3:** Association of demographic, preoperative, and postoperative data with outcome.

	Sustained Response *p*
***Demographic Data***	
Age (at time of splenectomy)	**0.034**
Race	0.24
Sex	0.32
***Preoperative Data***	
Platelets at diagnosis	0.43
Platelets at nadir	0.56
Splenomegaly	**0.018**
Fewer prior therapies	**0.0065**
Fewer relapses	0.060
Responsive to steroids and IVIG	0.51
***Postoperative Data***	
Length of Stay	0.19
Post-op platelet count (am next day)	0.27

## Data Availability

The data presented in this study are available on request from the corresponding author due to patient privacy considerations.
